# The association between self-management ability and malnutrition-inflammation-atherosclerosis syndrome in peritoneal dialysis patients: a cross-sectional study

**DOI:** 10.1186/s12882-020-02217-6

**Published:** 2021-01-07

**Authors:** Zehui Huang, Junyan Fang, Ahui Song, Yan Tong, Hai Deng, Shan Wei, Ouyang Ji, Chun Hu, Pu Li, Chunli Zhang, Yingli Liu

**Affiliations:** Department of Nephrology, Shanghai Ninth People’s Hospital, Shanghai Jiao Tong University School of Medicine, Shanghai, 200011 China

**Keywords:** Peritoneal dialysis, Self-management, Malnutrition, Inflammation, Atherosclerosis

## Abstract

**Background:**

The relationship between malnutrition-inflammation-atherosclerosis syndrome (MIAS) and self-management ability has not been previously revealed even though both play an important role in the management of peritoneal dialysis (PD) patients.

**Methods:**

In total, 93 patients were enrolled in this study. A self-management questionnaire was used for the evaluation of self-management ability. The identification of MIAS was based on one or more of the following three conditions: C-reactive protein (CRP)≥10 mg/L, malnutrition–inflammation score (MIS)> 7, and the presence of atherosclerosis-related medical records. The possible association between different self-management abilities and MIAS was analyzed with a Spearman correlation analysis.

**Results:**

There were 40 (43.0%) patients in the atherosclerosis group, and 38 (40.9%), 38 (40.9%), 10 (10.8%), and 7 (7.5%) patients in the MIAS0, MIAS1, MIAS2, and MIAS3 groups, respectively. The group with a score above the mean score of the Dialysis Effect Evaluation and Monitoring dimension had a fewer number of hospitalizations, higher albumin levels, lower MIS scores, a lower level of IL-6, and a lower number of MIAS factors. The Pearson and Spearman correlation analyses also revealed that this dimension was negatively correlated with the MIAS, MIS, IL-6, BNP, number of hospitalizations, and age and positively associated with albumin and prealbumin.

**Conclusion:**

The Dialysis Effect Evaluation and Monitoring dimension of the self-management scale for PD patients is closely linked to the MIAS, and a better dialysis effect evaluation and monitoring capacity results in a decreased likelihood of exposure to malnutrition and inflammation.

**Trial registration:**

Chinese Clinical Trial Registry: ChiCTR2000035525 (http://www.chictr.org.cn/showproj.aspx?proj=58110), registered August 13, 2020.

**Supplementary Information:**

The online version contains supplementary material available at 10.1186/s12882-020-02217-6.

## Background

As recommended by the International Society for Peritoneal Dialysis (ISPD) [1], peritoneal dialysis (PD) aims to provide high-quality patient-centered care. These care goals aim not only to maintain patients on PD to achieve their life goals, but also to ensure the provision of high-quality dialysis. PD is an operation performed at home without the supervision of medical staff, and the patient’s self-management ability is essential for achieving the adequacy of dialysis. The “adequacy of PD” assessment included symptoms, individual experiences and goals, residual kidney function, volume status, biochemical measures, nutritional status, cardiovascular function, small solute clearance and sense of well-being and satisfaction.

Patients or their caregivers receive pre-dialysis education, which includes PD-related knowledge and medicine-related knowledge training to improve self-management abilities, and after passing the training assessment, they can be approved to perform PD independently at home. This training course helps patients establish a concept of sterility and trains patients to follow aseptic techniques, monitor changes in their daily vital signs, obtain independent skills in PD fluid change operations and dialysis catheter care, identify unexpected conditions and seek PD center help [2, 3].

Strengthening the training of PD patients and improving their abilities can reduce the incidence of PD-associated peritonitis [4], enhance self-efficacy [5], and achieve a better fluid and nutritional status [6]. Although all patients received standardized training, there were differences in the self-management ability among the patients, leading to differences in PD outcomes.

The interaction between malnutrition and inflammation in dialysis patients is a key factor causingatherosclerosis to be easily overlooked [7]. The “reverse epidemiology” of malnutrition-inflammation-atherosclerosissyndrome (MIAS) refers to the opposite phenomenon of that observed in the general population, which has a high risk of cardiovascular disease due to the presence of a high body mass index (BMI) and hyperlipidemia [8]. Many studies have shown that atherosclerosis plays an increasingly important role in the occurrence of cardiovascular disease, which is the leading cause of death in dialysis patients [9]. The diagnosis of MIAS is based on the presence of one or more of the following three conditions: malnutrition, inflammation, and background atherosclerosis [10]. The presence of MIAS in dialysis patients is significantly associated with all-cause mortality, and all-cause mortality increases as the number of MIAS factors increases [11]. Unfortunately, there is still no reliable treatment for MIAS, and early prevention may bekey to addressing MIAS [12]. All factors of MIAS, working alone or combined, are important predictors of cardiovascular mortality in dialysis patients. In this study, malnutrition was assessed by the malnutrition-inflammation score (MIS), which has been shown to be a more comprehensive tool for assessing malnutrition in dialysis patients than the subjective global assessment (SGA) [13]. In addition to its prominent role in assessing malnutrition, the MIS is significantly related to inflammation and quality of life [14],and predicts the occurrence of infection and cardiovascular events in PD [15]. The correlation between the MIS and inflammation indicators was also verified in this study. Moreover, C-reactive protein (CRP) ≥10 mg/L was used as an index of inflammation in this study andis an independent predictor of cardiovascular mortality in dialysis patients. However, interleukin-6 (IL-6) was shownto be a better predictor in a recent study [16]; therefore, we collected both datapoints to achievea better comparison.

To the best of our knowledge, no trial has evaluated the correlation between self-management abilities and MIAS. In this study, we aimed to identify whether this correlation exists in PD patients. The results of this study can provide new ideas for the management of MIASand improve the prognosis of PD patients.

## Methods

Ninety-threepatients undergoing regular PD for more than 3 months at the Department of Nephrology, Ninth People’s Hospital, Shanghai Jiao Tong University School of Medicine from April 1, 2019 to November 1, 2019 met the inclusion criteria for the self-management ability and MIAS correlation study. Patients who did not meet the following criteria were excluded:1. age less than 18 years;2. duration of dialysis shorterthan 3 months; 3.cognitive dysfunction, malignant tumor or chronic infection, such as tuberculosis or hepatitis B; and 4.acute infection, severe cardio-cerebro vascular event history, surgery or traumawithin 1 month before the data collection. According to the exclusion criteria, we excluded 3 tumor patients and 2 pneumonia patients. The remaining patients met the inclusion criteria. Allpatients received the same predialysis patient education and postdialysis management at our center and agreed to participate in the study. We collected blood samples to assess serum CRP, albumin, prealbumin, creatine (Cr), cholesterol, triglycerides, calcium, phosphorus, potassium, serum intact parathyroid hormone (iPTH), serum ferritin, IL-6and tumor necrosis factor-α (TNF-α) upon admission to the hospital for the PD assessment during the study period. All laboratory measurements were carried out at the Ninth People’s Hospital Laboratory using standardized and automated methods. We also collected demographic and biochemical data, such asthe number of hospitalizations, diabetes, andperitonitis, which were diagnosed according to the recommendations of the ISPD when at least 2 of the following were present: (1) clinical features consistent with peritonitis, i.e., abdominal pain and/or cloudy dialysis effluent; (2) dialysis effluent white cell count> 100/μL or > 0.1*10^9^/L (after a dwelling time of at least 2 h) with > 50% polymorphonuclear; and (3) a positive dialysis effluent culture [3].

### Self-management scale for PD patients

The self-management scale for PD patientswas designed by our research team. The scale includes Medication Compliance (4 items), Dietary Management (4 items), Recognition of Dialysis Complications and Adequacy Evaluation (6 items), Dialysis Effect Evaluation and Monitoring (3 items), and Peritoneal Standardized Operation (6 items), for a ​​total of 5 dimensions and 23items.Each itemis scored on a 4-point Likert scoring (i.e., “Not at all”, “Basically okay”, “Mostly okay”, and “No problem” or “Unclear”, “Basic understanding”, “Moderate understanding”, and “Full understanding”, which correspond to 0, 1, 2, and 3 points, respectively). There are no reverse-scored items. The total score ranges from 0 to 69 points. The higher the score, the better the self-management ability. The total scale score is the sum of the items in each dimension. The purpose and significance of the survey were explained by researchers familiar with the questionnaire before the data were collected. The researchers assisted those who had difficulty reading. The initial scale included 26 items in 5 dimensions and collected data of 132 PD patients. After item analysis, the items of the initial scale were screened, and all items were statistically significant. After three rotations and layer-by-level exploratory factor analysis, 5 common factors were extracted, and 23 items were finally retained. The cumulative variance contribution rate was 69.799%, and the load of each item factor was > 0.45. The Cronbach α coefficient of the total scale was 0.930, and the half coefficient was 0.946.

The assessment of self-management ability is a tool used to determine whether patients are independent and can perform PD safely at home. As explained above, the standardization of the operation ability (such as standard sterility PD exchange, hand hygiene, and exit-site care) must be regularly monitored to avoid PD-related infection and identify changes in volume (such as blood pressure, weight, urine volume, and edema) to avoid fluid overload. We also need to monitor their diet management abilities to maintain a good nutritional status and medicine management abilities to avoid adverse drug reactions and identify PD-related complications (such as the identification of peritonitis symptoms, ductal dysfunction and inadequate dialysis symptoms) in a timely manner. All these evaluation indicators could directly affect the adjustment of the PD prescription.

However, there is no internationally standardized scale for the evaluation of the self-management abilities of PD patients. Xiaohua Wang et al. designed a continuous ambulatory PD patient self-management scale including 28 items and 5 dimensions, namely, solution bag replacement, troubleshooting during operation, diet management, complication monitoring, emotion management and return to social life [17]. To make it easier for the patients to understand and ensure that the content is more comprehensive, we deleted and added some content based on the existing scale. We simplified the troubleshooting during the operation dimension and complication monitoring dimension and combined these dimensions into the Dialysis Effect Evaluation and Monitoring dimension; the Recognition of Dialysis Complications and Adequacy Evaluation dimension was designed to replace the emotional management and return to social life dimension, andwe added the Medication Compliance dimension to evaluate the medication management ability. Our scale paid more attention to evaluating self-management consciousness during daily dialysis operations (see the appendix for details, Additional file 1).

### Assessment of MIAS

MIAS is a complex involving malnutrition, inflammation, and atherosclerosis, and MIAS is divided into MIAS0, MIAS1, MIAS2, and MIAS3 based on the presence of zero, one, two, and three components, respectively [7, 10]. We used the MIS to evaluate malnutrition. The score includes 4 aspects, including relevant medical history, physical examination, and laboratory indicators (BMI, plasma albumin, and transferrin). In total, there are 10 items; each item is scored from 0 to 3 to indicate the severity from mild to severe, and a higher total score indicates more severe malnutrition. A total score less than 7 indicates a well-nourished state, and a score greater than 7 indicates malnutrition. Inflammation was indicated by CRP≥10 mg/L because the normal range in this center is less than 10 mg/L. Atherosclerosis was defined as the presence of one of the following conditions: 1. a previous medical history of coronary heart disease, acute myocardial infarction, cerebral infarction, or cavity cerebral infarction; 2. cervical artery or lower limb artery ultrasound showing plaque formation; and 3. cranial computed tomography/magnetic resonance imaging (CT/MRI) and chest CT showing intracranial infarction or coronary calcification. Therefore, according to the answer “yes” regarding whether the MIS was > 7 points, CRP was ≥10 mg/L, and atherosclerosis-related history or examination results were present, the patients were divided into the MIAS0, MIAS1, MIAS2, and MIAS 3 groups. Patients with more than one MIAS factor were combined as MIAS (1–3).

### Statistical methods

After collecting the data, a dedicated researcher was responsible for the data input and verification and used EpiData3.1 to establish a database and SPSS23.0 (Chicago, IL, USA) for the statistical analysis. Student’s t-test, Mann-Whitney U test and χ^2^ test were used to compare the normally distributed, nonnormally distributed and categorical data, respectively. The continuous variables are presented as the mean±standard deviation, and the categorical variables are shown as frequencies with percentages. Pearson and Spearman correlation analyses were used to assess the correlations between the parametric and nonparametric data, respectively*.* Statistical significance was indicated by *P* values< 0.05.

## Results

### Demographical characteristics of the entire cohort

There were 51 (54.8%) males and 42 (45.2%) femaleswith a mean age of 60.66 ± 13.36 years. The causes of kidney failure were diabetic nephropathy in 27 (29%) patients, chronic nephritis in 14 (15.1%) patients, hypertensive renal injury in 4 (4.3%) patients, IgA nephropathy in 4 (4.3%) patients, polycystic kidney disease in 3 (3.3%) patients, and other or unknown in 41 (44.1%) patients. Seventeen patients were suffering from PD-related peritonitis (17.9%). The mean duration of PD in these patients was 31.65 ± 24.48 months, and the longest dialysis time was 144 months. The duration of dialysis referred to the period from the date of dialysis initiation to the collection of the self-management scale. The mean MIS was 5.92±3.93, and patients with an MIS greater than 7 accounted for 23.7% of the sample. There were 40 (43.0%) patients in the atherosclerosis group and 38 (40.9%), 38 (40.9%), 10 (10.8%), and 7 (7.5%) patients in the MIAS0, MIAS1, MIAS2, and MIAS3 groups, respectively.

### Self-management scale for PD patients

The comparison of the scores between MIAS0 and MIAS (1–3) is shown in Table 1. Finally, the Dialysis Effect Evaluation and Monitoring dimension showed marked differences between the MIAS (1–3) and MIAS0 (*p*< 0.05) groups. Although the total scale did not show statistical significance (*p*> 0.05), the MIAS0 group had a higher total score than the MIAS (1–3) group.

**Table 1:** Differences in scores on the self-management scale among PD patients

Variable	All patients (*n*=93)	MIAS0 (*n*=38)	MIAS (1–3) (*n*=55)	*P* value
**T**otal scale	47.5±12.1	49.7±12.5	46.0±11.7	0.150
**M**edication compliance	6.8±2.5	7.0±2.4	6.8±2.7	0.734
**D**ietary management	9.5±3.5	9.4±3.6	9.6±3.5	0.829
**R**ecognition of dialysis complication and adequacy evaluation	8.8±5.6	9.9±5.6	8.0±5.5	0.100
**D**ialysis effect evaluation and monitoring	5.6±2.4	6.6±2.3	5.4±2.4	**0.015**
**P**eritoneal standardized operation	16.2±3.0	16.2±3.1	16.2±2.9	0.951

Data are shown as the mean ± standard deviation; bold *P* value is statistically significant

*MIAS* Malnutrition-Inflammation-Atherosclerosis Syndrome

### Clinical and laboratory characteristics

In the self-management scale, the total scores of self-management and each dimension were grouped according to the mean. Statistically significant results were found in the Dialysis Effect Evaluation and Monitoring dimension group. For convenience, we defined the group with a score higher than the mean as Group 2 and the group with a score lower than the mean as Group 1. Group 2 had a fewer number of hospitalizations (7.76±4.74 vs. 11.55±7.70), better nutritional and inflammation status as indicated by higher albumin levels (33.89±4.37 vs. 31.05±4.54 g/L), lower MIS scores (4.51±2.49 vs. 7.97±4.69), higher hemoglobin levels (107.40±21.62 vs. 98.84±17.24 g/L), higher triglycerides levels (2.34±1.41 vs. 1.67±0.87 mmol/L), lower IL-6 levels (7.07±3.93 vs. 13.54±16.05 pg/mL), and a lower number of MIAS factors (49.09% vs. 73.68%) than Group 1 (*P*< 0.05) (Table 2).

**Table 2:** Clinical and biochemical characteristics of PD patients in this study (*n* = 93)

Variable	Group1※ ≤5 (*n*=38)	Group2※ >5 (*n*=55)	t/χ^2^	*P* value
Age (year)	63.0±13.1	59.1±13.4	1.397	0.166
Diabetes (n, %)	15 (39.5)	23 (41.8)	0.180	0.671
Male (n, %)	20 (52.6)	31 (56.4)	0.126	0.722
Atherosclerosis (n, %)	23 (60.5)	20 (36.4)	3.699	0.054
Peritonitis (n, %)	8 (21.1)	9 (16.4)	0.331	0.565
Dialysis duration (months)	36.4±26.5	28.4±22.6	1.576	0.118
Number of Hospitalizations	11.56±7.7	7.8±4.7	2.701	**0.009**
BMI (kg/m^2^)	21.7±3.1	23.5±2.7	−2.899	**0.006**
Serum albumin (g/L)	31.1±4.5	33.9±4.4	−3.092	**0.003**
Prealbumin (g/L)	0.3±0.1	0.4±0.1	−2.706	**0.009**
Creatinine (μmol/L)	886.6±275.6	911.7±322.6	−0.392	0.696
GFR (mL/min/1.73m^2^)	5.0±1.5	5.1±2.2	−0.388	0.699
Hemoglobin (g/L)	98.8±17.2	107.4±21.6	−2.033	**0.045**
Ferritin (μg /L)	255.5±274.2	181.0±181.3	1.468	0.147
Transferrin (g/L)	1.72±0.49	1.83±0.46	−1.089	0.279
iPTH (pmol/L)	326.6±267. 6	291.3±209.0	0.712	0.478
Calcium (mmol/L)	2.2±0.3	2.2±0.2	−1.167	0.246
Phosphorus (mmol/L)	1.7±0.8	1.7±0.4	0.131	0.896
Sodium (mmol/L)	140.6±3.7	139.7±7.3	0.673	0.502
Potassium (mmol/L)	3.9±0.8	3.9±0.7	0.071	0.943
Cholesterol (mmol/L)	4.3±1.1	4.5±1.3	−0.547	0.586
Triglycerides (mmol/L)	1.7±0.9	2.3±1.4	−2.810	**0.006**
CRP (mg/L)	13.7±22.9	5.9±14.8	1.855	0.069
IL-6 (pg/mL)	13.5±16.1	7.1±3.9	2.166	**0.038**
TNF-α (ng/mL)	16.8±4.5	15.9±9.6	0.566	0.630
BNP (pg/mL)	768.0±1171.6	275.7±437.6	2.474	**0.017**
MIS	8.0±4.7	4.5±2.5	4.163	**0.000**
MIAS0 (n, %)	10 (26.3)	28 (50.9)	5.625	**0.018**
MIAS1 (n, %)	14 (36.8)	24 (43.6)	0.429	0.512
MIAS2 (n, %)	8 (21.1)	2 (3.6)	6.667	**0.010**
MIAS3 (n, %)	5 (13.2)	1 (1.8)	5.077	**0.024**
MIAS (1–3) (n, %)	28 (73.7)	27 (49.1)	5.979	**0.014**

Data are shown as the mean ± standard deviation, number (percentage); bold *P* values are statistically significant

*BMI* Body mass index, *GFR* Glomerular filtration rate, *iPTH* Intact parathyroid hormone, *CRP* C-reactive protein, *IL-6* Interleukin-6, *TNF-α* Tumor necrosis factor-α, *BNP* B-type natriuretic peptide, *MIS* Malnutrition inflammation score, *MIAS* Malnutrition-inflammation-atherosclerosis syndrome;

※Group 2 and Group 1 are logograms for distinguishing scores above and below the Dialysis Effect Evaluation and Monitoring dimension mean score

We compared the differences between the MIAS0 and MIAS (1–3) groups and found that MIAS0 patients were younger and had fewer hospitalizations, higher albumin and prealbumin levels, lower CRP levels, and a higher Dialysis Effect Evaluation and Monitoring dimension score. Binary logistic regression analysis was performed, and the results showed that age was the only independent risk factor for MIAS. Age is an irreversible risk factor for atherosclerosis, which may explain why it is an independent risk factor for MIAS.

### Pearson and Spearman correlation analysis results

After the Pearson and Spearman correlation analysis of each dimension of the self-management scale with the MIS and MIAS, only the Dialysis Effect Evaluation and Monitoring dimension had a statistically significant and negative correlation with the MISand MIAS (Table 3). Based on the above results, we conducted a correlation analysis between the biochemical parameters and Dialysis Effect Evaluation and Monitoring dimension. This dimension was significantly associated with IL-6, the number of hospitalizations, BNP, age, albumin, and prealbumin **(**Table 4**)**.

**Table 3:** Correlation analysis results of the self-management scale with MIS and MIAS

Variable	MIS		MIAS	
	R	P	R	P
**T**otal scale	− 0.100	0.342	−0.152	0.146
**P**eritoneal standardized operation	− 0.068	0.516	− 0.057	0.584
**D**ialysis effect evaluation and monitoring	−0.342	**0.000**	−0.257	**0.013**
**M**edication compliance	0.107	0.305	−0.011	0.914
**D**ietary management	−0.050	0.634	−0.025	0.841
**R**ecognition of dialysis complications and adequacy evaluation	0.021	0.998	−0.048	0.645

Bold *P* values are statistically significant; *MIS* Malnutrition inflammation score, *MIAS* Malnutrition-inflammation-atherosclerosis syndrome

**Table 4:** Correlation analysis results of the Dialysis Effect Evaluation and Monitoring dimension with demographic and biochemical variables in PD patients

Variable	Dialysis effect evaluation and monitoring capacity	
	R	P
MIS	−0.342	0.000
MIAS	−0.257	0.013
IL-6	−0.347	0.001
Albumin	0.341	0.001
Prealbumin	0.207	0.046
BNP	−0.249	0.016
Age	−0.243	0.019
Number of hospitalizations	−0.222	0.033

*IL-6* Interleukin-6, *BNP* B-type natriuretic peptide, *MIS* Malnutrition inflammation score, *MIAS* Malnutrition-inflammation-atherosclerosis syndrome

Previous studies have shown that the MIS is a powerful tool for the diagnosis of protein-energy wasting (PEW) [18]. Notably, the MIS was also found to be correlated with the laboratory indicators predicting malnutrition and inflammation in this study. The MIS was positively correlated with age, the number of hospitalizations, the presence of atherosclerosis, ferritin, IL-6, TNF-α, CRP and MIAS and inversely correlated with the BMI, albumin, prealbumin, hemoglobin, transferrin, creatinine and blood phosphorus (Table 5).

**Table 5:** Correlation of MIS with demographic and biochemical variables in PD patients

Variable	MIS	
	R	P
Age	0.329	0.001
BMI	−0.354	0.001
Number of hospitalizations	0.272	0.008
Presence of atherosclerosis	0.297	0.004
Albumin	−0.626	0.000
Prealbumin	−0.399	0.000
Hemoglobin	−0.256	0.014
Creatinine	−0.211	0.042
Ferritin	0.452	0.000
Transferrin	−0.275	0.008
Phosphorus	−0.220	0.034
IL-6	0.280	0.015
TNF-α	0.364	0.000
CRP	0.358	0.000
MIAS	0.682	0.000

*BMI* Body mass index, *CRP* C-reactive protein, *IL-6* Interleukin-6, *TNF-α* Tumor necrosis factor-α, *MIS* Malnutrition inflammation score, *MIAS* Malnutrition-inflammation-atherosclerosis syndrome

## Discussion

To the best of our knowledge, this study is the first to reveal the correlation between self-management ability and MIAS in patients undergoing PD. We quantified self-management through a homemade self-management scale and found that the Dialysis Effect Evaluation and Monitoring dimension was closely linked to MIAS, which was divided into four groups based on the presence of zero, one, two, or three of the following components: MIS> 7, CRP≥10 mg/L, and background atherosclerosis; the better this capacity, the better the malnutrition and inflammation status. The total number of patients who had three components of MIAS accounted for 7.5% of the sample.

After completing the self-management ability survey, we found that the awareness of the standard operation was acceptable; however, the knowledge of dialysis-related complications and dialysis adequacy evaluation was weak. This finding provides new ideas for the enrichment of the patient education content in the future.

A Japanese study found that the combination of more MIAS factors will result in a stronger the predictive power for the 36-month all-cause mortality [11]. This study also found that 41.9% of the patients had atherosclerosis, which is worthy of considering to avoid further deterioration and improve prognosis. Currently, a targeted treatment is lacking, and screening for malnutrition and inflammation in an early stage and then adopting some effective steps to intervene immediately are critical [7, 19]. The present study demonstrated that a close relationship exists between the Dialysis Effect Evaluation and Monitoring dimension and MIAS; meanwhile, the higher the score, the lower the chance of having a component of MIAS. The reasons for this correlation can be explained by the following aspects. The patient-centered self-management program assists patients in following prescriptions and maintaining better nutritional and volume status [6]. Karadag E et al. found that improving the self-management capacity has been shown to help reduce the occurrence of PD-related peritonitis [4, 5]. A patient’s outstanding self-management abilitycannot be separated from the patient’s positive and optimistic attitude towards life;moreover, depression and cognitive declineproved to be closely linked to MIAS in PD patients [20]. To date, research concerning self-management ability and MIAS or individual factors alone is limited. Our study may be a pilot study, and further studies exploring the causality among the factors are warranted.

As this study reveals, the Dialysis Effect Evaluation and Monitoring dimension is closely linked with the MIS, which has been shown to be a more comprehensive tool than the subjective global assessment (SGA) in diagnosing PEW [21]. It is worth noting that with regard to malnutrition in dialysis patients, we should pay attention to distinguishing between malnutrition and PEW. Malnutrition refers to insufficient dietary intake or inability to meet human needs because of dietary restrictions or anorexia, whereas PEW is more likely to refer to decreased protein and energy storage due to various chronic kidney disease (CKD)-related factors, such as uremia toxins, inflammation and a high metabolic state [22]. Thus, PEW may be more accurate in describing malnutrition in dialysis patients and the ideal MIAS component. However, there is no uniform cutoff value for the MIS. Mariana et al. conducted a cohort study to set a reasonable cutoff value for the MIS to better predict the prognosis of hemodialysis patients and found that anMIS higher than 7 was a reasonable cutoff for predicting mortality [23], andthe same result was found in predialysis patients [24]. This study also verified that the MIS has a good correlation with the malnutrition indicators hemoglobin and prealbumin and inflammation indicators CRP, IL-6, TNF-a and ferritin in PD patients (Table 5). Ultimately, we found that 23.7% of the patients were exposed to malnutrition when including MIS> 7 as an MIAS-compliant criterion. Notably, the prevalence of malnutrition deserves further attention, and it is important to further identify a rational MIS cutoff value for PD patients to assess malnutrition and allow for precautions to be taken in a timely manner.

According to the mean total score and each dimension, group comparisons were performed. In the Dialysis Effect Evaluation and Monitoring dimension group, patients with scores above the mean had lower MIS scores and higher albumin levels. Furthermore, the inflammation indictors, such as IL-6, were lower in this group. Chronic inflammatory states are commonly found in patients with end-stage renal disease [25], and inflammatory cytokines, including IL-6, IL-1, TNF-a and CRP play an important role [7]. Notably, here, inflammation refers to an increase in pro-inflammatory factors rather than infection caused by microorganisms [26]. The causes of inflammation are complex, and strong links with malnutrition and cardiovascular disease are involved in mortality and adverse cardiovascular outcomes [7]. CRP, as an acute time-response protein, is an important predictor of cardiovascular mortality, but Le Viet Thang et al. found that IL-6 is a better predictor of 5-year cardiovascular mortality [16]. As previous studies have shown, a higher self-management ability means that the patient has a better nutritional status and volume status [6], and a lower likelihood of exposure to PD-related peritonitis [5]. These factors are all possible causes of the prevalence of inflammation in dialysis patients, which makes the results of this study reasonable.

The correlation analysis between the Dialysis Effect Evaluation and Monitoring dimension and clinical data revealed that this dimension was negatively correlated with the number of hospitalizations and age and positively associated with prealbumin. Thisfinding suggests that improving the ability of self-monitoring of PD, to some extent, can help reduce the number of hospital admissions and then indirectly reduce medical expenses. In addition, although the comparison of the dialysis duration was not statistically significant (*P*=0.118), it appeared to exhibit a downward trend. In other words, patients’ self-managementabilities may decline with longer dialysis. Therefore, the importance of providing additional dialysis-related knowledge is emphasized, which has also been confirmed in some studies that demonstrated that retraining can help reduce the incidence of PD-associated peritonitis and improve self-efficacy [4, 5].

In the future, the health education of PD patients should pay more attention to increasing knowledge of dialysis complications and adequacy and improving daily monitoring capabilities. Targeted patient self-management education programs should be based on different ages and dialysis durations to increase patient awareness of recording urine output, monitoring blood pressure and weight changes, and seeking timely help in the event of discomfort. Thus, high-quality dialysis patient education includes not only strengthening standardized dialysis operations to prevent infections, thereby reducing the incidence of PD-associated peritonitis, but also teaching patients more about how to improve their s0elf-monitoring capabilities and the ability to recognize complications, such as keeping a good record of dialysis diaries, recognizing uncomfortable symptoms and seeking medical support in a timely manner. Future longitudinal studies could also further clarify the causal relationship between MIAS and self-monitoring to allow early measures to be adopted to prevent the occurrence of MIAS.

### Limitations

We recognize several deficiencies in this study. First, the single-center cross-sectional study design may compromise the credibility of the results. Second, even though a strong correlation was observed between MIAS and self-management ability, a cohort study or randomized control study is needed to compare the changes in MIAS before and after the intervention of self-management ability and further reveal the causality between these factors. Third, there is no uniform reasonable MIS cutoff value, and the use of an MIS greater than 7 to indicate a malnutrition status as an MIAS comorbidity deserves further confirmation. Fourth, the scale used in this research was in Chinese version. In order to show the results of the research to colleagues, the scale was translated into English and polished by native language speaker. However, the linguistic validation of the scale used in this study may need to be performed to cover language barrier and more feedback is expected to improve the scale.

## Conclusions

This cross-sectional study revealed that the higher the score on the Dialysis Effect Evaluation and Monitoring domain of the self-management scale for PD patients, the better the nutritional status. In addition, the negative correlation between self-management ability and MIAS requires longitudinal studies to further confirm the causal relationship between the two factors. Thus, this study provides a new idea for the treatment and management of MIAS and highlights the importance of improving self-management abilities.

## Supplementary Information

**Additional file 1.**

## Data Availability

All data generated or analyzed during this study are included in this published article and its supplementary information files.

## Abbreviations

MIAS: Malnutrition-inflammation-atherosclerosis syndrome
PD: Peritoneal dialysis
CRP: C-reactive protein
MIS: Malnutrition–inflammation score
ISPD: International Society for Peritoneal Dialysis
BMI: Body mass index
SGA: Subjective global assessment
IL-6: Interleukin-6
BNP: Type B natriuretic peptide
Cr: Creatine
iPTH: Intact parathyroid hormone
TNF-α: Tumor necrosis factor-α
CT/MRI: Computed tomography/magnetic resonance imaging
PEW: Protein-energy wasting
