# Supplementary material for: The association between self-management ability and malnutrition-inflammation-atherosclerosis syndrome in peritoneal dialysis patients: a cross-sectional study
Source: BMC Nephrol. 2021 Jan 7;22:13. doi: 10.1186/s12882-020-02217-6 (PMC7791726; doi:10.1186/s12882-020-02217-6)
Supplement: Supplementary file 1 — Additional file 1. [file 12882_2020_2217_MOESM1_ESM.docx]

**Self-management scale for peritoneal dialysis patients**

Objective:

|  | 0 | 1 | 2 | 3 |
| --- | --- | --- | --- | --- |
| **1. Peritoneal Standardized Operation** | | | | |
| Can you do the fluid replacement by yourself or relatives and nanny who are also trained? |  |  |  |  |
| Can you keep the fluid replacement environment clean, ventilated and dry? |  |  |  |  |
| Can you wash your hands with soap and wear clean gloves when changing the iodophor?  Can you clean the catheter port and the skin next to it regularly? |  |  |  |  |
| Can you change the fluid on time and register every ultrafiltration in the record book? |  |  |  |  |
| Can you strictly follow the daily dialysis plan made by your doctor for fluid replacement? |  |  |  |  |
| **2. Dialysis Effect Evaluation and Monitoring** | | | | |
| Can you weigh yourself every morning? |  |  |  |  |
| Can you measure your blood pressure every day? |  |  |  |  |
| Can you make regular outpatient follow-up? |  |  |  |  |
| **3. Medication Compliance** | | | | |
| Can you accurately say the type of oral drugs you are currently taking? |  |  |  |  |
| Can you accurately say the number and time of oral drugs you are taking? |  |  |  |  |
| Can you accurately distinguish the effects of current oral drugs? |  |  |  |  |
| Can you take your medicine regularly and on time? |  |  |  |  |
| **4. Dietary Management** | | | | |
| Can you take the initiative to understand which symptoms indicate that you currently have calcium and potassium deficiency, high phosphorus or malnutrition, etc.? |  |  |  |  |
| Can you reduce your water intake based on ultrafiltration and weight changes? |  |  |  |  |
| Can you be able to take the initiative to understand the dietary knowledge suitable for patients with renal failure? |  |  |  |  |
| Can you guide your daily diet based on dietary knowledge from medical care or online? |  |  |  |  |
| **5. Recognition of Dialysis Complications and Adequacy Evaluation** | | | | |
| Do you know the adverse effects of unregulated operations? |  |  |  |  |
| Do you know the symptoms that suggest peritoneal dialysis-related peritonitis? |  |  |  |  |
| Do you know what conditions indicate that the operation failed? |  |  |  |  |
| Do you know the significance of monitoring changes in blood pressure and weight? |  |  |  |  |
| Do you know what indicators should be monitored for each outpatient visit? |  |  |  |  |
| Do you know the standard of peritoneal dialysis adequacy? |  |  |  |  |
| **Total score** | | | | |

Each item is scored on a 4-point Likert scoring "No at all", "Basically okay", "Most okay", "No problem " , or "Unclear", "Basic understanding", "Most understanding", and "Full understanding" which correspond to 0, 1, 2, and 3 points respectively.
